# Supplementary material for: DeepTRACE brings flexible machine learning to single-molecule track analysis
Source: Commun Biol. 2026 Apr 14;9:812. doi: 10.1038/s42003-026-09899-y (PMC13265943; doi:10.1038/s42003-026-09899-y)
Supplement: Supplementary file 1 — Supplementary Information [file 42003_2026_9899_MOESM1_ESM.pdf]

Supplementary Information
DeepTRACE brings flexible machine learning to single-molecule track analysis

Oliver J Pambos[*,1,2], Jacob AR Wright[1,2], and Achillefs N Kapanidis[1,2]

[1]Clarendon Laboratory, Department of Physics, University of Oxford, Parks Road, Oxford, OX1 3PU, United Kingdom

[2]Kavli Institute for Nanoscience Discovery, University of Oxford, Sherrington Road, Oxford, OX1 3QU, United Kingdom

[*]Correspondence: oliver.pambos@physics.ox.ac.uk

Supplementary Figures

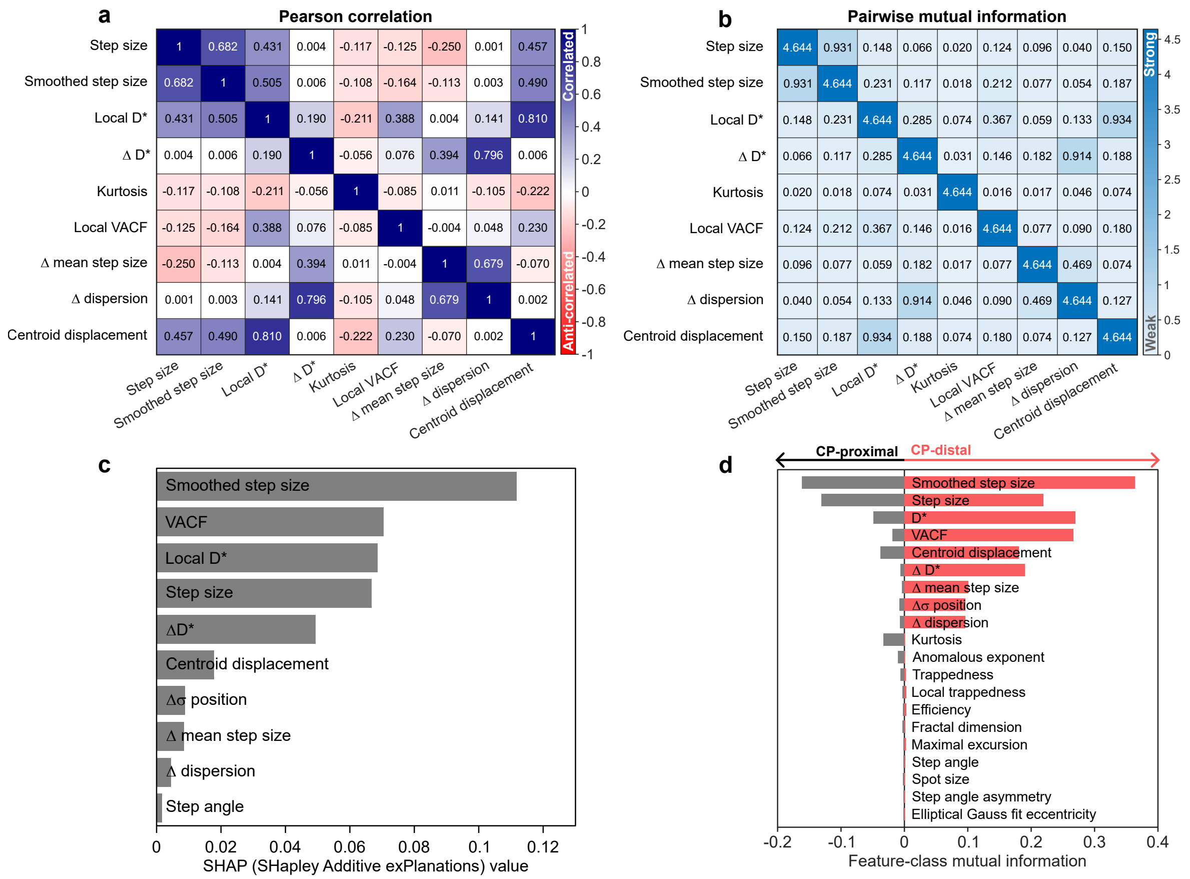

Supplementary Figure 1: DeepTRACE feature relationships and ranking. **a**, Linear relationships between an example subset of features revealed by Pearson correlation coefficients (r) between pair-wise combinations. **b**, Non-linear relationships between a subset of features, revealed by a mutual information map between pairwise feature combinations. **c**, SHAP (**SH**apley **A**dditive ex**P**lanation) values for features evaluated using a surrogate random forest model trained in DeepTRACE. **d**, Ranked mutual information between a subset of features and known class, providing a prediction of the relative static power of features to discriminate classes, in both changepoint-proximal (defined here as the four localisations flanking each changepoint; grey bars, left) and changepoint-distal regions (red bars, right). All plots were produced using DeepTRACE's *Feature Ranking* tool from n = 1,253 independent tracks from two-state reversible diffusion simulations ($D = 0.01$ and $0.05\,\mu\text{m}^2/\text{s}$) using ground truth for class assignment.

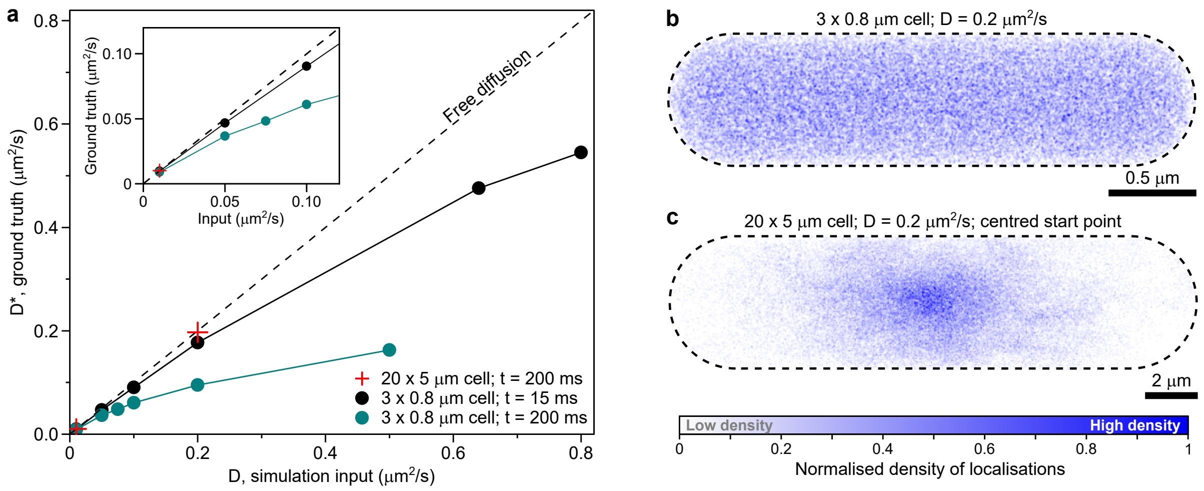

Supplementary Figure 2: Anomalous sub-diffusion arises from cellular confinement. **a**, Apparent diffusion coefficients obtained from DeepTRACE's *Diffusion Analysis* tool of simulated intracellular particle motion computed using MSD calculations of ground truth-segmented subtracks representing pure diffusive states; showing subdiffusive behaviour at both frame rates used (teal circles represent timelapse imaging at intervals of 200 ms, black circles represent interframe times of 15 ms) resulting from confinement as molecules are influenced by the boundary. The dashed line represents free diffusion with an anomalous exponent of $\alpha = 1$. Red crosses represent a control simulation showing greatly reduced anomalous sub-diffusion when particles are generated starting at the centre of an expanded $20\,\mu\text{m} \times 5\,\mu\text{m}$ rod-shaped cell, illustrating the impact of confinement. **b-c**, Heatmap reconstructions of particle locations for all fast state track segments ($D = 0.2\,\mu\text{m}^2/\text{s}$) using DeepTRACE's *Spatial Mapping* tool with subtrack boundaries obtained from ground truth. **b**, All localisations from the fast diffusive state subtracks of $n = 1{,}234$ independent molecules simulated within a $3\,\mu\text{m} \times 0.8\,\mu\text{m}$ rod-shaped cell, with uniformly distributed starting locations, showing a high degree of interaction with the enclosing cell membrane. The spatial map was constructed by positioning point spread functions (PSFs) with a full-width half maximum (FWHM) of 10 nm at each localisation and rendering into an image with a pixel scale of 5 nm. **c**, All localisations from the fast diffusive state subtracks of $n = 490$ independent tracks simulated within an expanded ($20\,\mu\text{m} \times 5\,\mu\text{m}$) rod-shaped cell, with all molecules starting at the cell centre, showing a greatly reduced probability of interaction with the cell membrane. The spatial map was constructed using PSFs with FWHM of 50 nm rendered onto an image with a pixel scale of 25 nm.

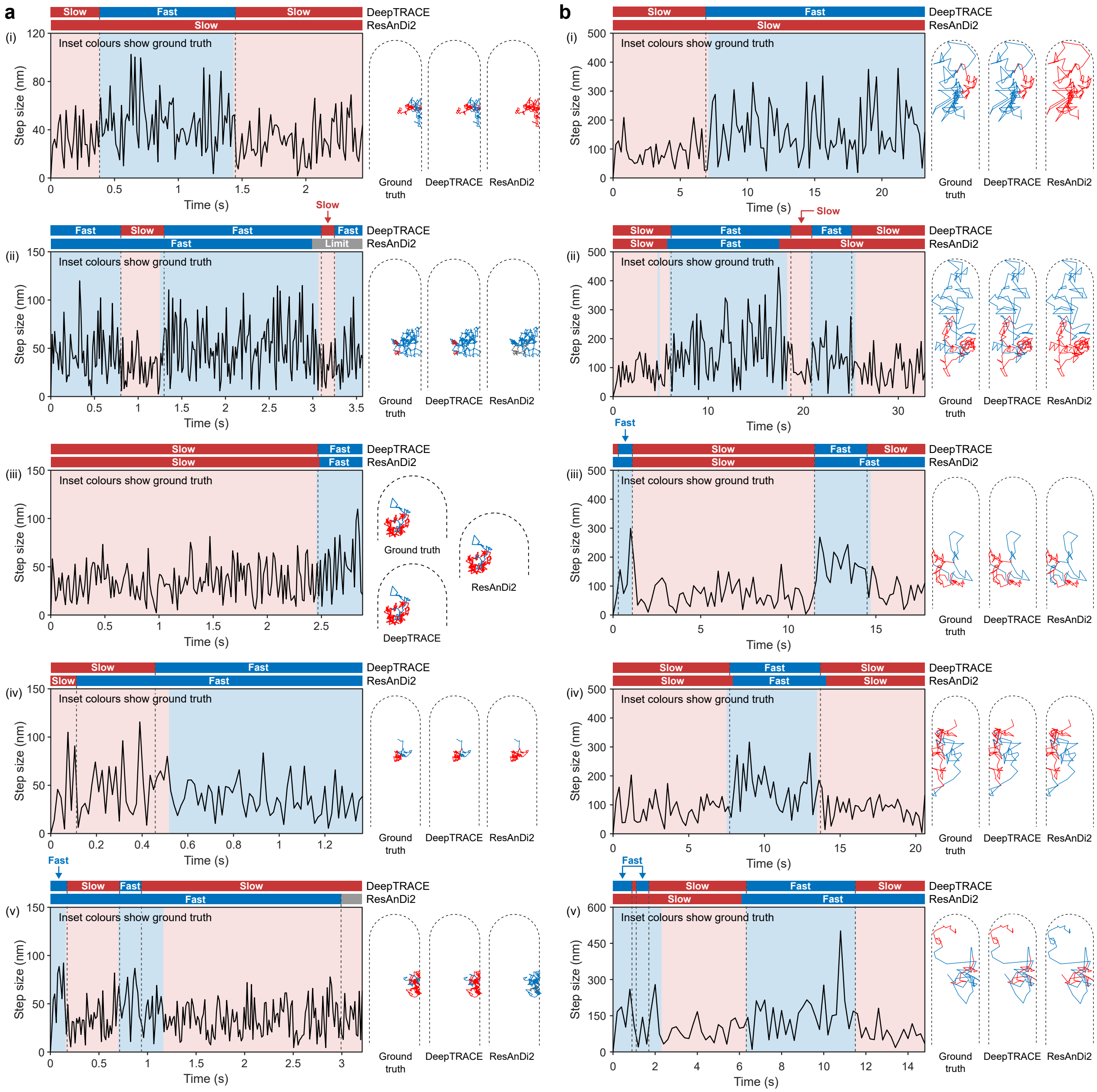

Supplementary Figure 3: Example classifications of challenging two-state diffusion simulations. **a**, Classifications for tracks generated with diffusion coefficients of $D_{\mathrm{slow}} = 0.01\,\mu\mathrm{m}^2/\mathrm{s}$ and $D_{\mathrm{fast}} = 0.05\,\mu\mathrm{m}^2/\mathrm{s}$ at interframe times of 15 ms, common in trackingPALM experiments. Classifications were performed by a 4,817-parameter DeepTRACE model trained on $n = 147$ independent tracks. **b**, The same simulations performed under timelapse imaging with interframe times of 200 ms. Classifications were performed by a 2,172-parameter DeepTRACE model trained on $n = 1{,}065$ independent tracks. The bars above each plot show classifications by a DeepTRACE model and ResAnDi2, while inset colours display ground truth. To the right of each time series are plotted single-molecule tracks coloured by classification. Tracks were selected using the random track selector of DeepTRACE's *Track Inspector* tool, discarding any tracks which did not possess changepoints. Example tracks were selected prior to inclusion of ResAnDi2 in the comparative analysis.

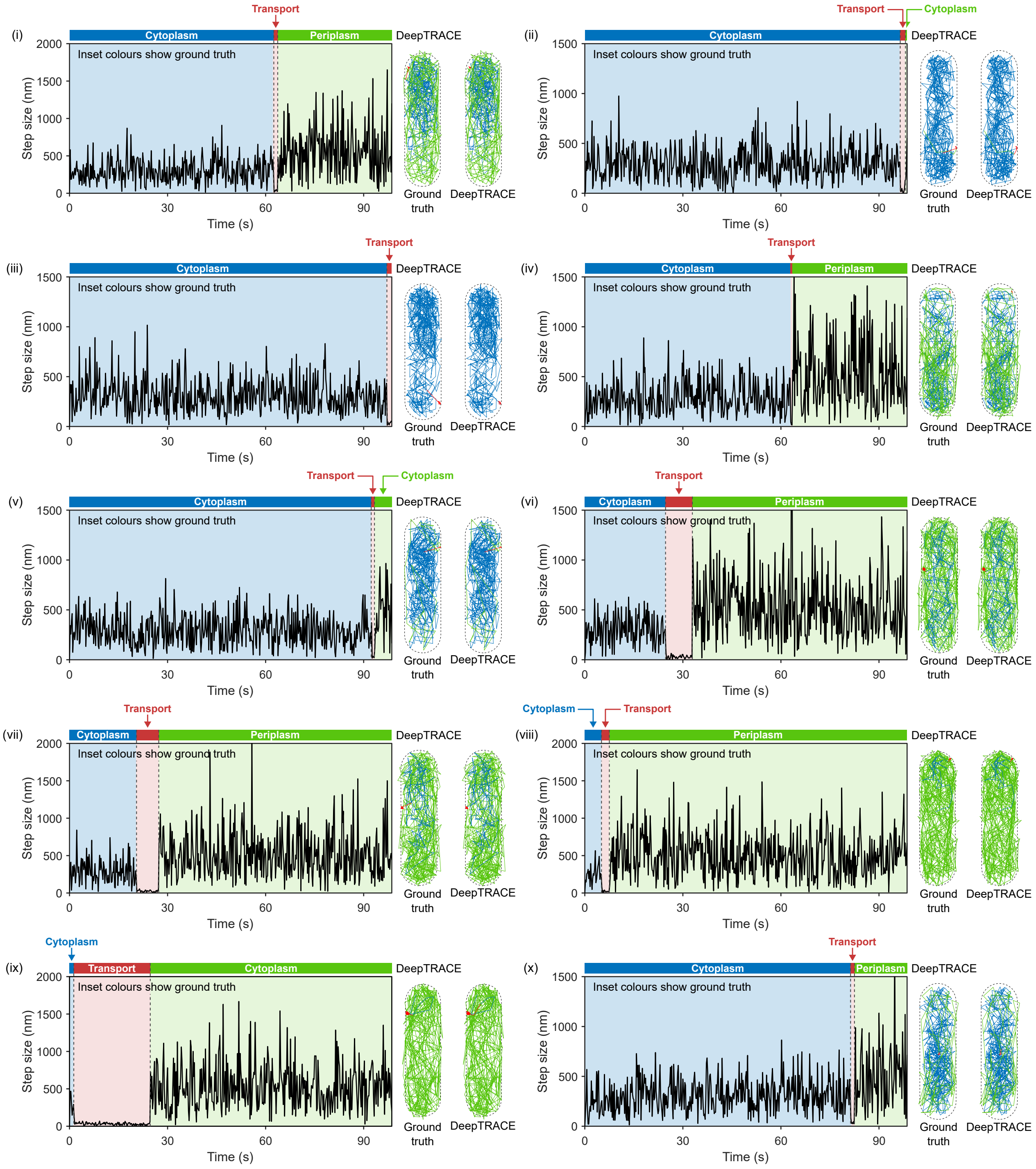

Supplementary Figure 4: Example classifications of simulations of irreversible transport across the cytoplasmic-periplasmic membrane. Classifications by a 2,273-parameter DeepTRACE model trained on $n = 1{,}298$ independent simulated tracks are shown above each time series plot, while inset colours show ground truth. To the right are the plotted single molecule tracks within the model cell boundary (dashed black lines). Localisations falling outside of the model cell boundary arise from localisation uncertainty, common in tracking applications (e.g. localisation error).

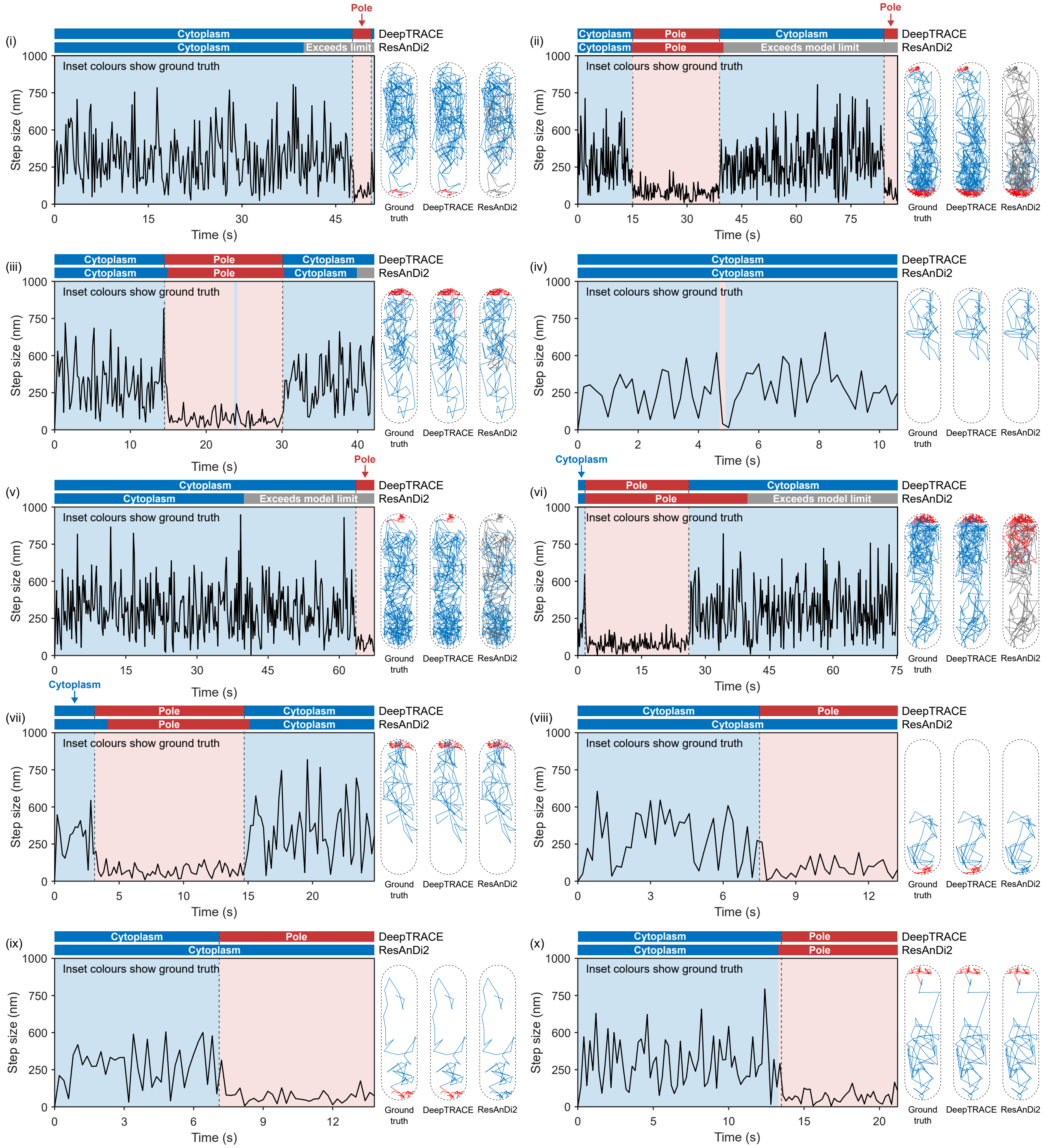

Supplementary Figure 5: Example classifications of polar-localised plasmid binding simulations. Classifications by a 2,252-parameter DeepTRACE model trained on n = 1,061 independent simulated tracks, and ResAnDi2 are shown in the annotation bar above each time series plot, while inset colours show ground truth. Shown to the right of each time series are the plotted tracks within the cell boundary (dashed black line), coloured by classification. Tracks were selected randomly by DeepTRACE's *Track Inspector* tool, discarding those which did not possess changepoints in the ground truth. Example tracks were selected prior to inclusion of ResAnDi2 in the comparative analysis.

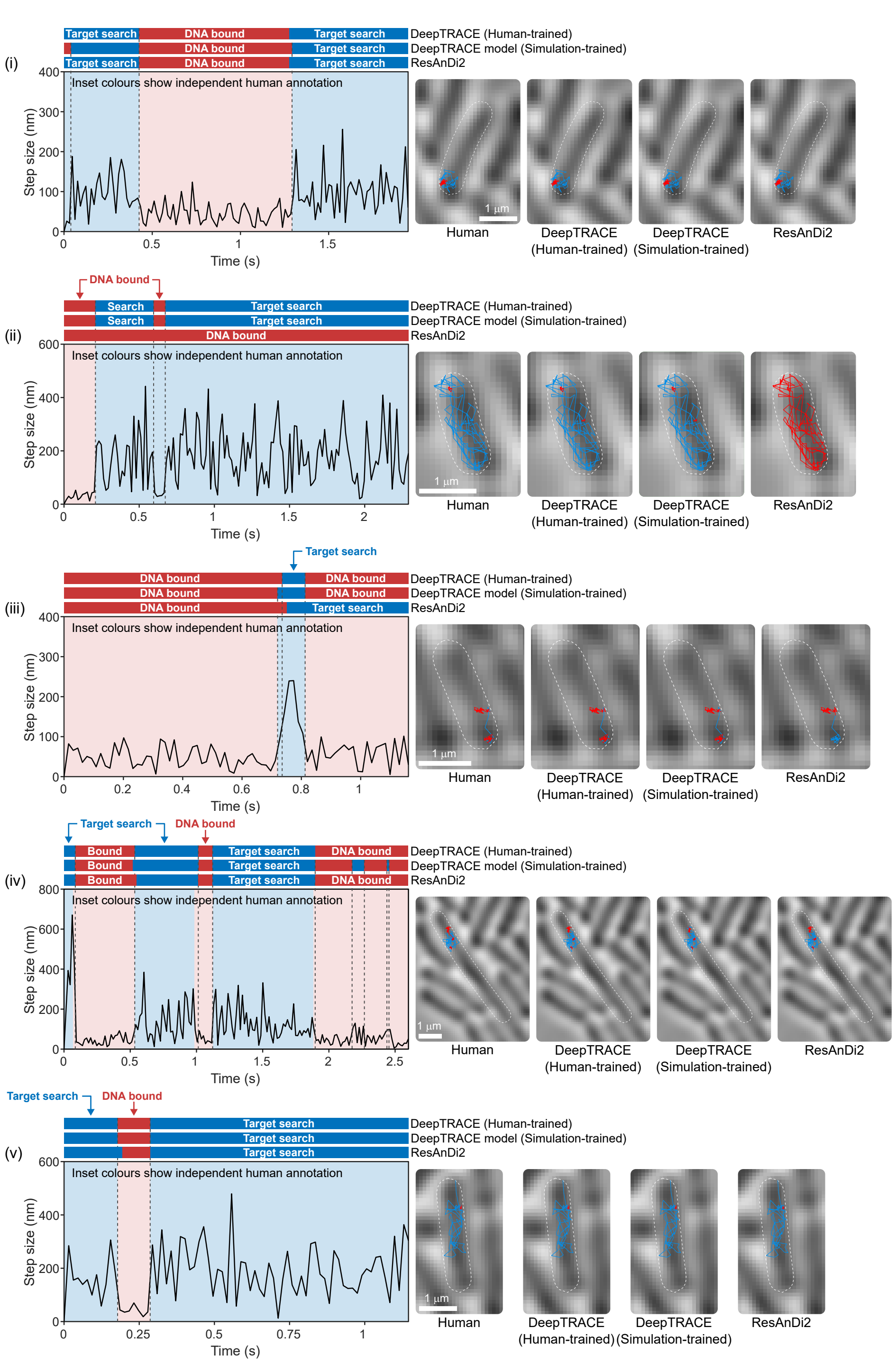

Supplementary Figure 6: Example classifications of real experimental DNA Polymerase I experiments. Inset colours show independent experienced human classifications. Bars above show model classifications for: a 4,577-parameter DeepTRACE model trained on n = 453 human annotations from a separate experiment, a 4,577-parameter DeepTRACE model trained on n = 1,098 independent simulated tracks, and ResAnDi2. Right: plotted tracks coloured by classification, overlaid onto the inverted brightfield reference image, with segmented cell boundary (dashed white line). Tracks were selected randomly by DeepTRACE's *Track inspector* tool, discarding those which did not possess changepoints in the human classification. Example tracks were selected prior to inclusion of ResAnDi2 in the comparative analysis.

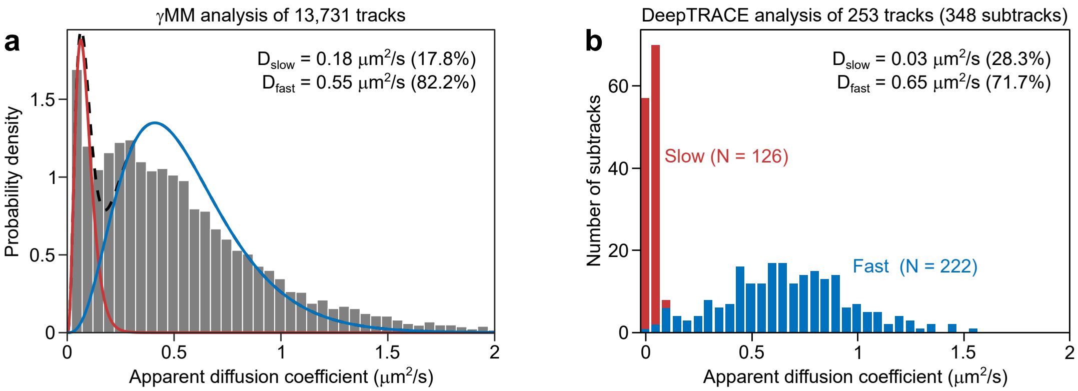

Supplementary Figure 7: Improvement of diffusive state separation through track segmentation. Comparison using real single-molecule tracking experimental data of DNA repair by DNA polymerase I (Pol1) in *E. coli* recorded using 15 ms continuous imaging, five minutes after H_2O_2-induced DNA damage[1]. **a**, Diffusion histogram of tracking data compiled from MSD-lag time calculations of n = 13,731 Pol1 tracks truncated to the first five localisations, and analysed using a typical two-component Gamma mixture model (γMM) analysis[2,3], showing the two fitted Gamma functions (red and blue solid lines) and their sum (dashed black line). **b**, A subset of n = 253 tracks selected from the same single field of view dataset by DeepTRACE based on track length and quality control criteria, segmented by a model into n = 348 subtracks, and compiled into a stacked histogram by DeepTRACE's *Diffusion Analysis* tool, illustrating improved state discrimination of both states, closely matching established values[1]. The model was trained on n = 869 human-annotated tracks from separate experiments under similar experimental conditions.

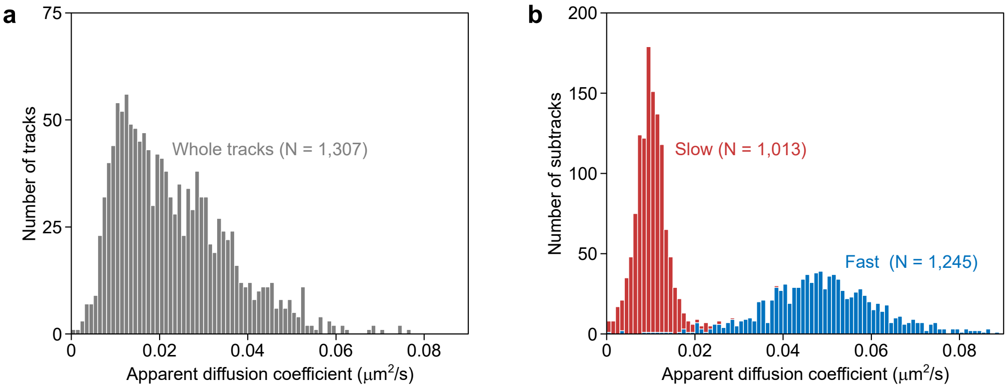

Supplementary Figure 8: Accurate state refinement via DeepTRACE segmentation in a two-state diffusion simulation. **a**, Histogram of apparent diffusion coefficients (D^*) computed from $n = 1{,}307$ independent whole tracks via MSD–lag time analysis, shows the merging of slow ($0.01\,\mu\text{m}^2/\text{s}$) and fast ($0.05\,\mu\text{m}^2/\text{s}$) populations. **b**, Stacked histogram of apparent diffusion coefficients using the same analysis applied to the same set of tracks following segmentation by a 4,848-parameter DeepTRACE model trained on 147 independent tracks into $n = 1{,}013$ slow and $n = 1{,}245$ fast subtracks (red and blue columns respectively), reveals clear separation into two peaks closely matching the simulated diffusion coefficients. The data was obtained from a two-state diffusion simulation generated with an interframe time of 15 ms.

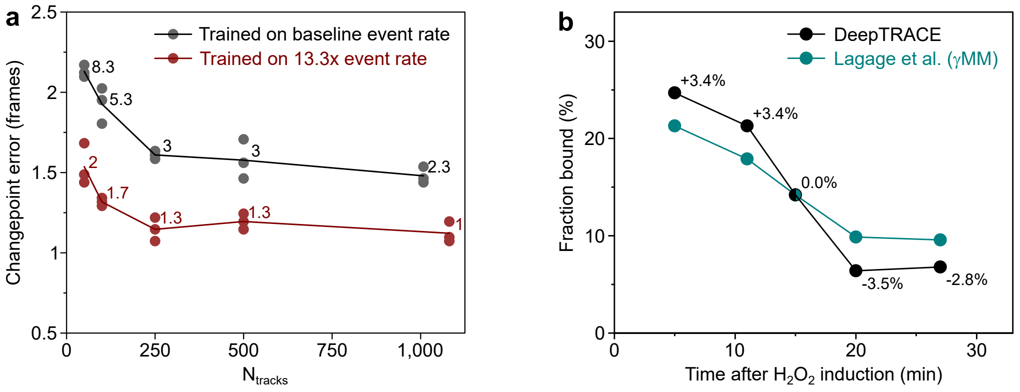

Supplementary Figure 9: Training models using experimental perturbations in simulated and real experiments. a, Performance comparison of changepoint detection in reversible two-state diffusion simulations, between models trained on data with class transition rates matching the evaluated dataset (0.1 Hz and 0.05 Hz, black circles), and models trained with data containing a perturbation enhancing rates to 1.33 Hz and 0.67 Hz (red circles). The changepoint error is the mean number of frames separating the predicted and known changepoints from the simulation ground truth (see Methods). The evaluated dataset contained $n = 188$ independent tracks with 43 changepoints. Individual points display the changepoint error for $n = 3$ independently trained models for each dataset size, each using an independently randomised training/validation split and shuffled batches, with lines showing the mean. Data labels show the mean number of unmatched changepoints when compared to ground truth. Both training datasets were fully independent of the data used for evaluation; with each downsampled subset ($n \leq 500$ independent tracks) obtained by a separate resampling of the full simulation by random track elimination (from $n = 1{,}186$ and $n = 1{,}272$ independent tracks in the original baseline and enhanced simulations respectively). **b**, Fraction of time spent by DNA polymerase I molecules in the DNA-bound state during a timecourse series of real single-molecule tracking experiments following exposure to the DNA-damaging agent H_2O_2, as evaluated by a model trained on $n = 453$ independent human-annotated tracks from a separate experiment 4 min following H_2O_2 exposure (black circles), compared to estimates obtained from γMM analysis of the same recordings as reported by Lagage et al.[1] (teal circles). While no ground truth is available for real experimental data, both analyses reflect the expected reduction in bound state occupancy over time. Data labels indicate the difference in bound population between estimates from DeepTRACE and γMM.

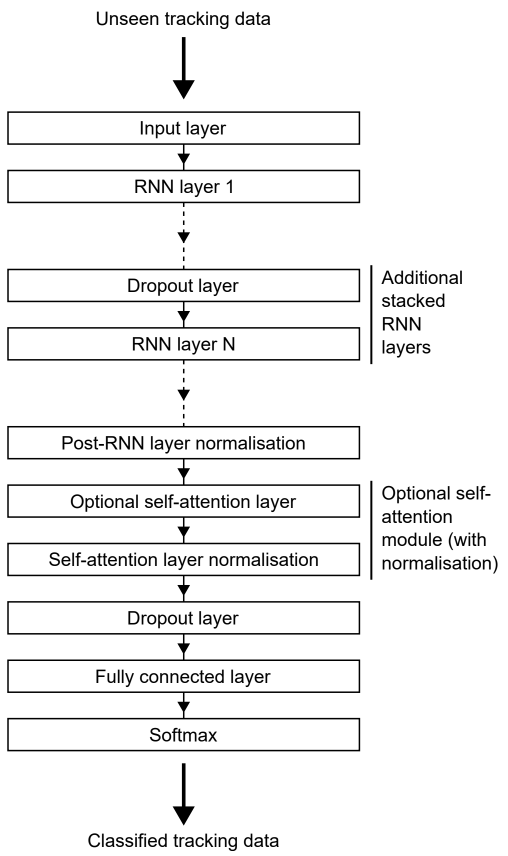

Supplementary Figure 10: Modular architecture for RNN-based models. RNN-based DeepTRACE models can be constructed by the user through the GUI using a standardised model architecture based on a deep sequence model design incorporating an arbitrary number of stacked RNN layers, and optional self-attention. The model architecture performs sequence-to-sequence classification of unseen data.

Supplementary Tables

Supplementary Table 1: Performance comparison of estimated kinetic parameters from $n =$ 1,292 independent simulated tracks using a DeepTRACE model trained on ground truth labels from $n = 167$ independent tracks from a separate simulation (**Sim-GT**), a DeepTRACE model trained on human annotations of the same $n = 167$ independent tracks (**Sim-Human**), and a DeepTRACE model trained on human annotations of $n = 453$ tracks from real experiments of DNA polymerase I (Pol1) repairing H_2O_2-induced DNA damage (**Real-Human**), with the same $n =$ 1,292 independent simulated tracks evaluated using the popular software **Spot-On**, a Gamma mixture model (**γMM**), and **ResAnDi2**. Tracks were generated using a reversible two-state diffusion simulation with an interframe time of 15 ms, and diffusion coefficients of 0.01 $\mu m^2/s$ and 0.64 $\mu m^2/s$, matching reported Pol1 mobilities in *E.coli*[1]. The discrepancy between the ground truth apparent diffusion coefficient and the simulation input values, which is most prominent in the fast state, arises primarily from cellular confinement. The highest performing result for each parameter is highlighted in bold. All training data were generated independently from the dataset used for evaluation.

Parameter	Simulation input	Ground truth	DeepTRACE Sim-GT	DeepTRACE Sim-Human	DeepTRACE Real-Human	Spot-On	γMM	ResAnDi2
Slow state occupancy (%)	66.7	67.9	**68.1**	68.5	**68.1**	60.7	62.8	58.7
D^*_{slow} ($\mu m^2/s$)	0.0100	0.0098	**0.0105**	**0.0105**	0.0107	0.000	0.021	0.0207
D^*_{fast} ($\mu m^2/s$)	0.6400	0.5017	0.5117	**0.5050**	0.5145	0.412	0.387	0.4217
Slow → Fast transition rate (Hz)	0.667	0.654	**0.649**	**0.649**	0.684	N/A	N/A	0.984
Fast → Slow transition rate (Hz)	1.333	1.352	1.304	**1.379**	1.243	N/A	N/A	1.048

References

[1] Lagage, V., Chen, V. & Uphoff, S. Adaptation delay causes a burst of mutations in bacteria responding to oxidative stress. *EMBO reports* **24**, e55640 (2023).

[2] Stracy, M. *et al.* Live-cell superresolution microscopy reveals the organization of RNA polymerase in the bacterial nucleoid. *Proceedings of the National Academy of Sciences* **112** (2015).

[3] Uphoff, S., Reyes-Lamothe, R., Garza De Leon, F., Sherratt, D. J. & Kapanidis, A. N. Single-molecule DNA repair in live bacteria. *Proceedings of the National Academy of Sciences* **110**, 8063–8068 (2013).
