# Supplementary figures and images for: DeepTRACE brings flexible machine learning to single-molecule track analysis
Source: Commun Biol. 2026 Apr 14;9:812. doi: 10.1038/s42003-026-09899-y (PMC13265943; doi:10.1038/s42003-026-09899-y)

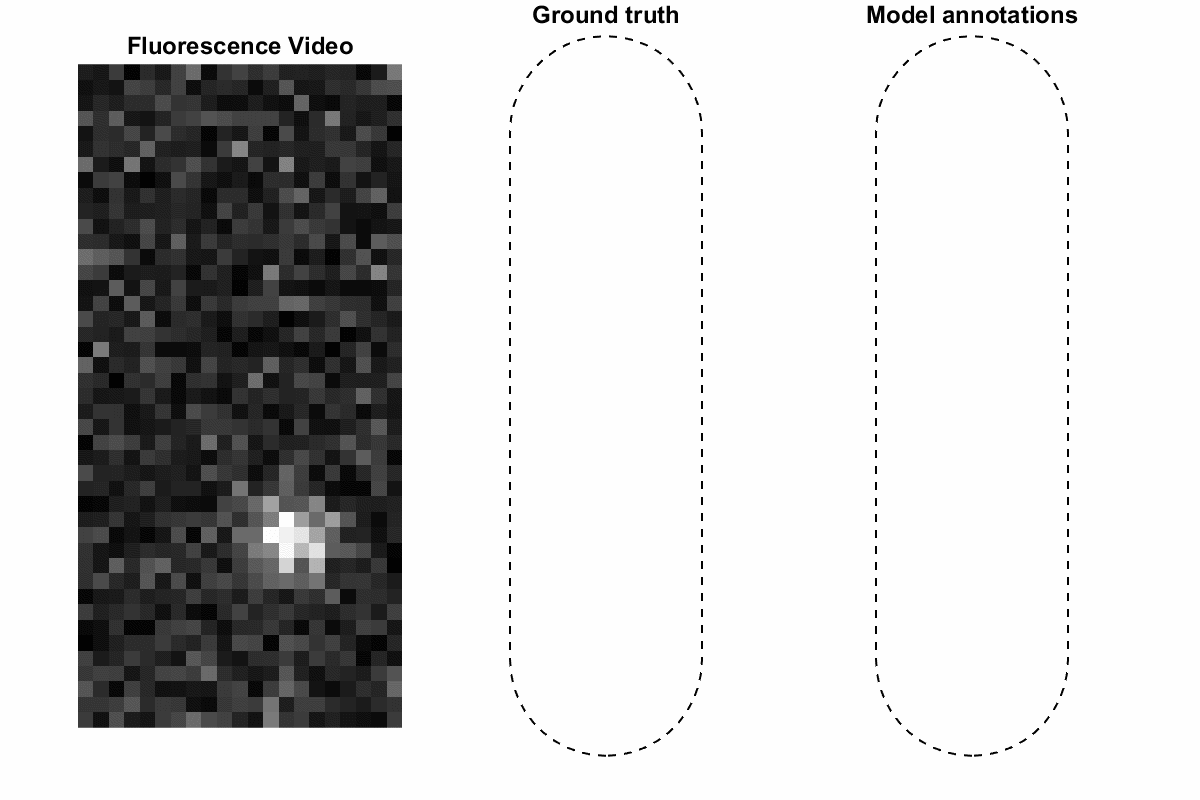

Supplement: Supplementary file 4 — Supplementary Movie 1 [file 42003_2026_9899_MOESM4_ESM.gif]

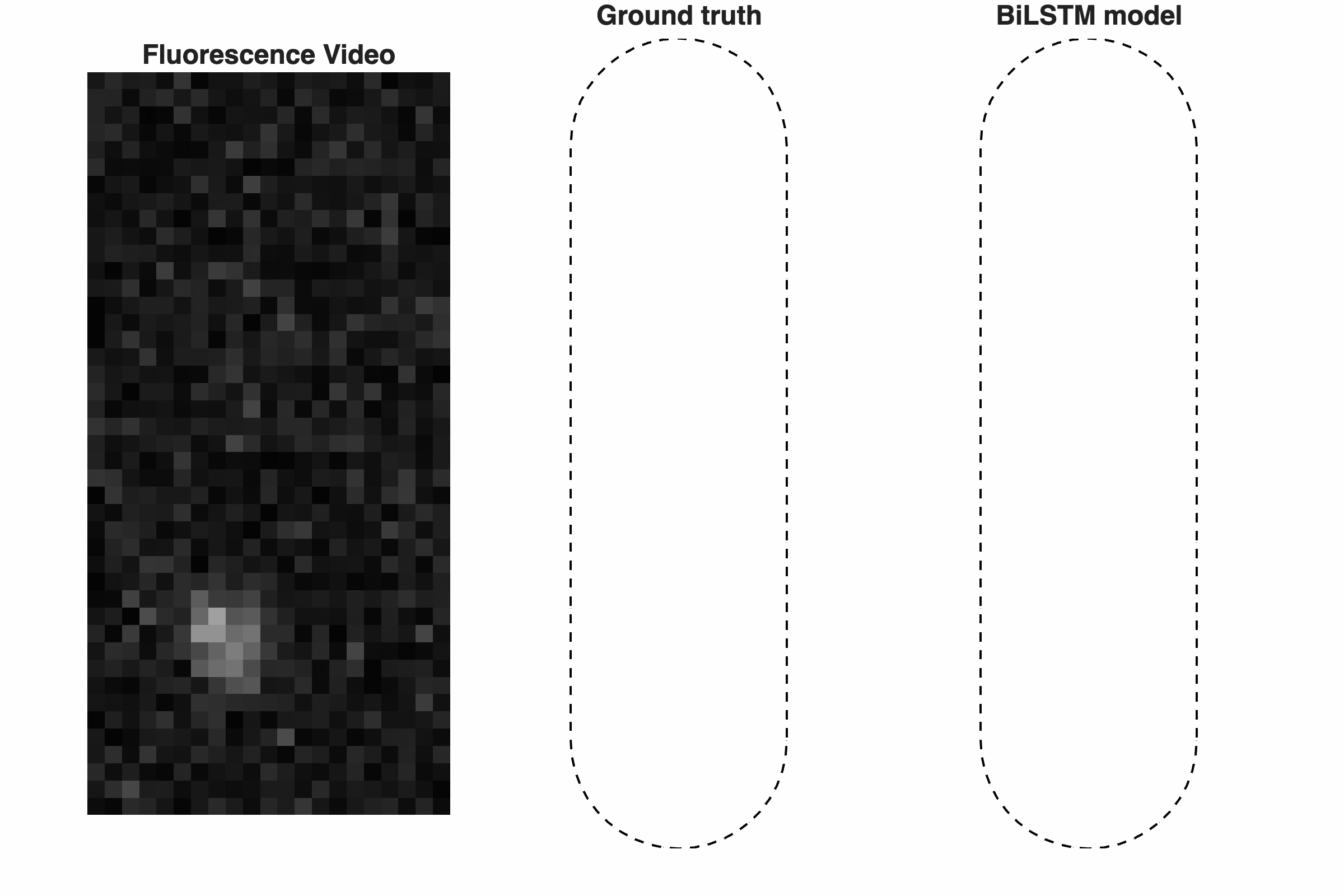

Supplement: Supplementary file 6 — Supplementary Movie 3 [file 42003_2026_9899_MOESM6_ESM.gif]

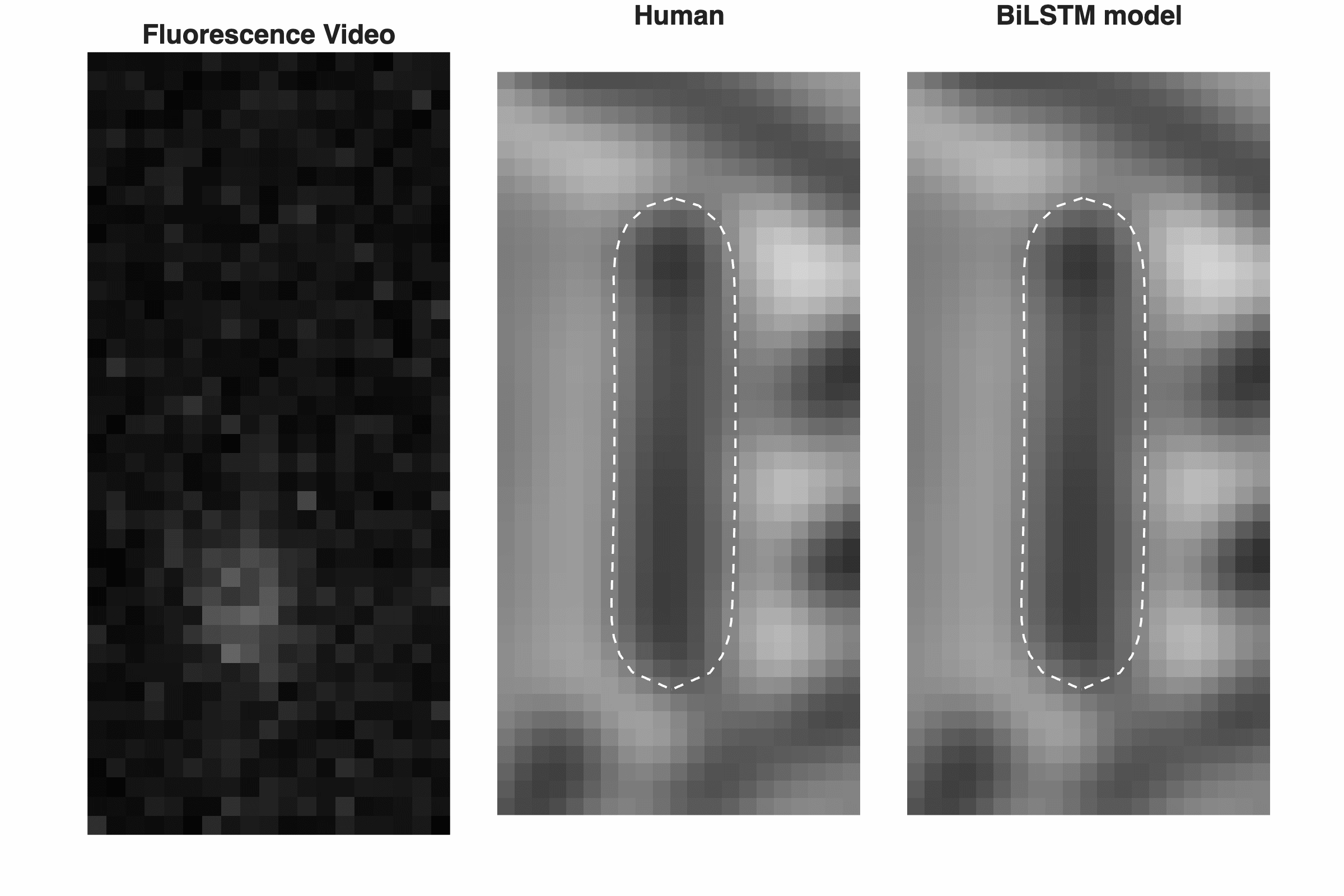

Supplement: Supplementary file 7 — Supplementary Movie 4 [file 42003_2026_9899_MOESM7_ESM.gif]

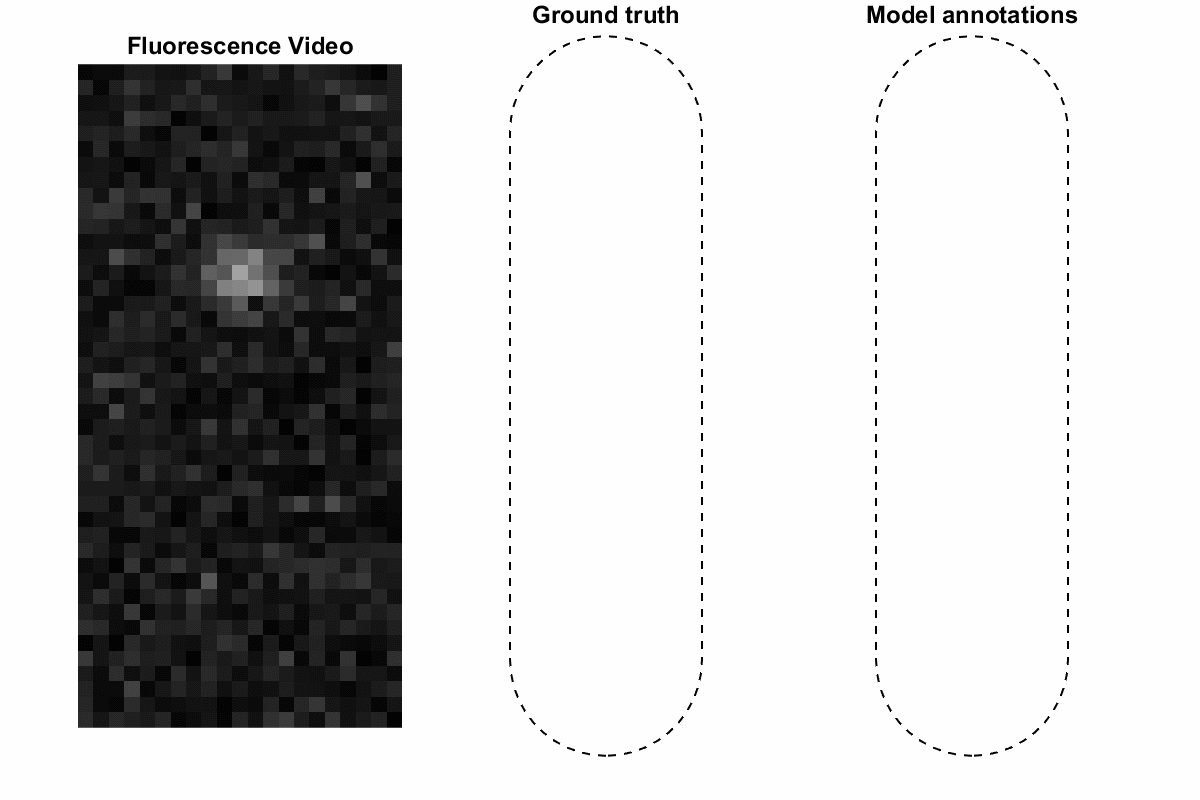

Supplement: Supplementary file 8 — Supplementary Movie 5 [file 42003_2026_9899_MOESM8_ESM.gif]

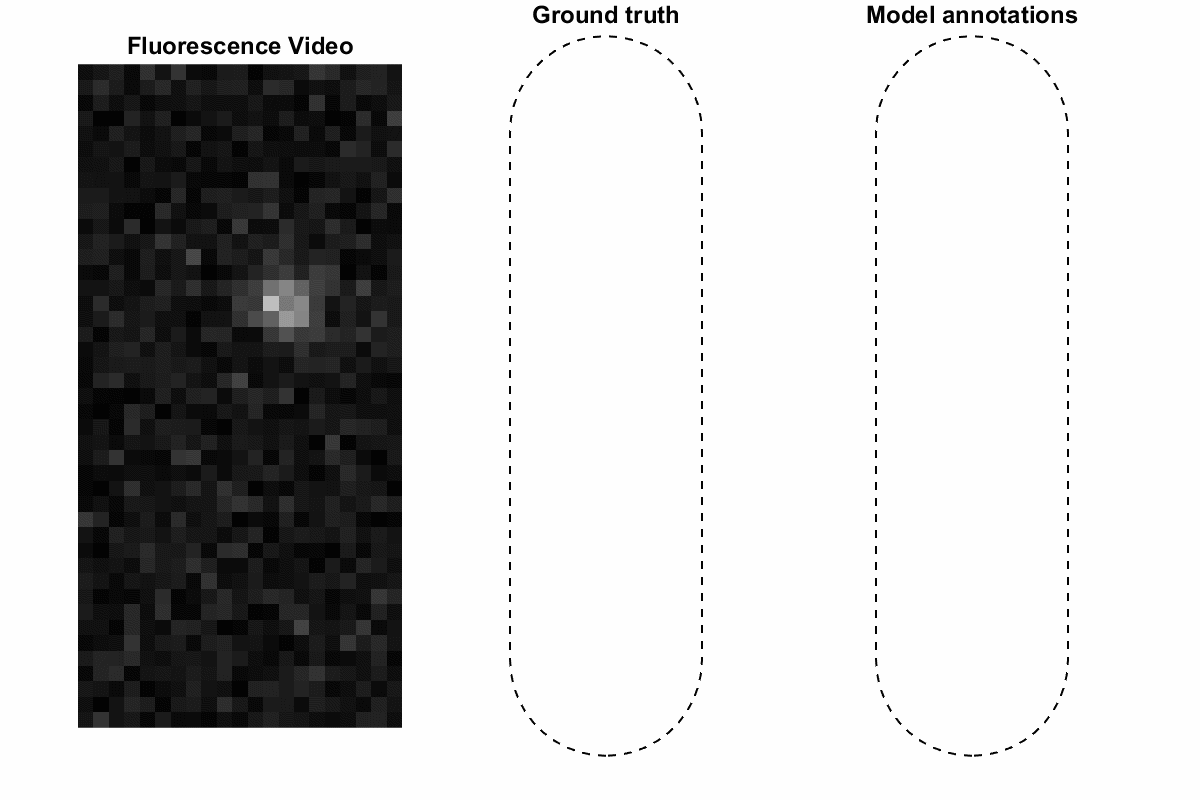

Supplement: Supplementary file 9 — Supplementary Movie 6 [file 42003_2026_9899_MOESM9_ESM.gif]
