# Supplementary material for: DeepTRACE brings flexible machine learning to single-molecule track analysis
Source: Commun Biol. 2026 Apr 14;9:812. doi: 10.1038/s42003-026-09899-y (PMC13265943; doi:10.1038/s42003-026-09899-y)
Supplement: Supplementary file 10 — Description of Additional Supplementary Files [file 42003_2026_9899_MOESM10_ESM.pdf]

# Description of Additional Supplementary Files

**File name:** Supplementary Data 1

**Description:** Source data for all data-containing figures

**File name:** Supplementary Data 2

**Description:** A list of the core engineered features implemented in the current version of DeepTRACE used in this study. Additional features undergoing development and testing will be incorporated in future releases.

**File name:** Supplementary Movie 1

**Description:** Synchronized fluorescence video and model annotation corresponding to the reversible two state diffusion simulation shown in Fig. 3b. The left panel shows the raw fluorescence sequence of the tracked molecule; the centre panel shows the reconstructed track coloured by the ground truth state; and the right panel shows the track coloured by predictions made by a DeepTRACE model trained on 147 independent tracks. The dynamic range is normalised independently for each frame.

**File name:** Supplementary Movie 2

**Description:** Synchronized fluorescence video and model annotation corresponding to the periplasmic transport simulation shown in Fig. 4a. The left panel shows the raw fluorescence sequence of the tracked molecule; the centre panel shows the reconstructed track coloured by the ground truth state; and the right panel shows the track coloured by DeepTRACE model predictions trained on 1,298 independent tracks from a separate simulation. The video displays the full sequence of frames, whereas Fig. 4a shows a cropped segment centred on the transport event for visual clarity. Video dynamic range is normalised globally across all frames.

**File name:** Supplementary Movie 3

**Description:** Synchronized fluorescence video and model annotation corresponding to the polar plasmid binding simulation shown in Fig. 5a. The left panel shows the raw fluorescence sequence of the tracked molecule; the centre panel shows the reconstructed track coloured by the ground truth state; and the right panel shows the track coloured by DeepTRACE model predictions trained on 1,061 independent tracks from a separate simulation. Video dynamic range is normalised globally across all frames.

**File name:** Supplementary Movie 4

**Description:** Synchronized fluorescence video and annotations corresponding to the single-molecule DNA repair event shown in Fig. 3d (Pol1 experimental data). The left panel shows the raw fluorescence sequence of the tracked molecule overlaid on an inverted brightfield reference image; the centre panel shows the reconstructed track coloured by independent human annotation; and the right panel shows the trajectory coloured by DeepTRACE model predictions using a model trained on 1,098 tracks from a two state

reversible diffusion simulation. Video dynamic range is normalised globally across the video. Dashed white lines indicate the segmented cell boundary.

**File name:** Supplementary Movie 5

**Description:** Synchronized fluorescence video and model annotation corresponding to the uniform binding simulation shown in Fig. 3f. The left panel shows the raw fluorescence sequence of the tracked molecule; the centre panel shows the reconstructed track coloured by the ground truth state; and the right panel shows the track coloured by DeepTRACE model predictions trained on 1,048 independent simulated tracks undergoing uniformly-distributed binding events. Video dynamic range is normalised globally across all frames.

**File name:** Supplementary Movie 6

**Description:** Synchronized fluorescence video and model annotation corresponding to the polar binding simulation shown in Fig. 3f. The left panel shows the raw fluorescence sequence of the tracked molecule; the centre panel shows the reconstructed track coloured by the ground truth state; and the right panel shows the track coloured by DeepTRACE model predictions trained on 1,048 independent simulated tracks undergoing uniformly-distributed binding events. Video dynamic range is normalised globally across all frames.
