# Supplementary material for: DeepTRACE brings flexible machine learning to single-molecule track analysis
Source: Commun Biol. 2026 Apr 14;9:812. doi: 10.1038/s42003-026-09899-y (PMC13265943; doi:10.1038/s42003-026-09899-y)
Supplement: Supplementary file 11 — Reporting Summary [file 42003_2026_9899_MOESM11_ESM.pdf]

Corresponding author(s): Oliver J. Pambos

Last updated by author(s): Sep 3, 2025

Reporting Summary

Nature Portfolio wishes to improve the reproducibility of the work that we publish. This form provides structure for consistency and transparency in reporting. For further information on Nature Portfolio policies, see our Editorial Policies and the Editorial Policy Checklist.

Statistics

For all statistical analyses, confirm that the following items are present in the figure legend, table legend, main text, or Methods section.

n/a	Confirmed	
☐	☒	The exact sample size (n) for each experimental group/condition, given as a discrete number and unit of measurement
☐	☒	A statement on whether measurements were taken from distinct samples or whether the same sample was measured repeatedly
☐	☒	The statistical test(s) used AND whether they are one- or two-sided<br>*Only common tests should be described solely by name; describe more complex techniques in the Methods section.*
☒	☐	A description of all covariates tested
☒	☐	A description of any assumptions or corrections, such as tests of normality and adjustment for multiple comparisons
☐	☒	A full description of the statistical parameters including central tendency (e.g. means) or other basic estimates (e.g. regression coefficient) AND variation (e.g. standard deviation) or associated estimates of uncertainty (e.g. confidence intervals)
☒	☐	For null hypothesis testing, the test statistic (e.g. F, t, r) with confidence intervals, effect sizes, degrees of freedom and P value noted<br>*Give P values as exact values whenever suitable.*
☒	☐	For Bayesian analysis, information on the choice of priors and Markov chain Monte Carlo settings
☒	☐	For hierarchical and complex designs, identification of the appropriate level for tests and full reporting of outcomes
☒	☐	Estimates of effect sizes (e.g. Cohen's d, Pearson's r), indicating how they were calculated

Our web collection on statistics for biologists contains articles on many of the points above.

Software and code

Policy information about availability of computer code

Data collection	Simulated data were produced using a custom pipeline incorporating Smoldyn version 2.72 for particle simulations, and video frame rendering with SMeagol (Lindén et al., 2016, Bioinformatics, https://doi.org/10.1093/bioinformatics/btw109). Previously-published experimental Pol1 single-molecule tracking datasets were kindly provided by Lagage et al. (2022, EMBO Reports, https://doi.org/10.15252/embr.202255640).
Data analysis	Single-molecule localisation (elliptical Gaussian fitting), track linkage, and gamma mixture model analysis were performed using the custom MATLAB pipeline described in Stracy et al. (2015, PNAS, https://doi.org/10.1073/pnas.1507592112). All downstream analyses were performed using DeepTRACE, as described in the main text and Online Methods, and will be made publicly available upon acceptance via an online repository. A snapshot of a minimal version of the codebase, it's GUI, sample data, a detailed walkthrough guide, and example pretrained models have been supplied to peer-reviewers for the purpose of evaluation and reproduction of results. Cell segmentation masks in the MicrobeTracker format for the real Pol1 experimental data were provided by Lagage et al. (2022, EMBO Reports, https://doi.org/10.15252/embr.202255640).

For manuscripts utilizing custom algorithms or software that are central to the research but not yet described in published literature, software must be made available to editors and reviewers. We strongly encourage code deposition in a community repository (e.g. GitHub). See the Nature Portfolio guidelines for submitting code & software for further information.

Data

Policy information about availability of data

All manuscripts must include a data availability statement. This statement should provide the following information, where applicable:

- Accession codes, unique identifiers, or web links for publicly available datasets
- A description of any restrictions on data availability
- For clinical datasets or third party data, please ensure that the statement adheres to our policy

Datasets containing single-molecule simulations used in the manuscript for key claims have been provided as part of the demonstration package for peer-reviewers and hosted by Zenodo. The editors have been provided with a private link and access code to share with reviewers. On publication the final version of the code, incorporating any modifications arising from peer review, together with the test data will be made available via a stable online repository. The real Pol1 experimental data was provided kindly by Lagage et al., as such we direct the reader to this publication for further information (2022, EMBO Reports, https://doi.org/10.15252/embr.202255640) as we are not the copyright holder.

Research involving human participants, their data, or biological material

Policy information about studies with human participants or human data. See also policy information about sex, gender (identity/presentation), and sexual orientation and race, ethnicity and racism.

Reporting on sex and gender	*Use the terms sex (biological attribute) and gender (shaped by social and cultural circumstances) carefully in order to avoid confusing both terms. Indicate if findings apply to only one sex or gender; describe whether sex and gender were considered in study design; whether sex and/or gender was determined based on self-reporting or assigned and methods used. Provide in the source data disaggregated sex and gender data, where this information has been collected, and if consent has been obtained for sharing of individual-level data; provide overall numbers in this Reporting Summary. Please state if this information has not been collected. Report sex- and gender-based analyses where performed, justify reasons for lack of sex- and gender-based analysis.*
Reporting on race, ethnicity, or other socially relevant groupings	*Please specify the socially constructed or socially relevant categorization variable(s) used in your manuscript and explain why they were used. Please note that such variables should not be used as proxies for other socially constructed/relevant variables (for example, race or ethnicity should not be used as a proxy for socioeconomic status). Provide clear definitions of the relevant terms used, how they were provided (by the participants/respondents, the researchers, or third parties), and the method(s) used to classify people into the different categories (e.g. self-report, census or administrative data, social media data, etc.) Please provide details about how you controlled for confounding variables in your analyses.*
Population characteristics	*Describe the covariate-relevant population characteristics of the human research participants (e.g. age, genotypic information, past and current diagnosis and treatment categories). If you filled out the behavioural & social sciences study design questions and have nothing to add here, write "See above."*
Recruitment	*Describe how participants were recruited. Outline any potential self-selection bias or other biases that may be present and how these are likely to impact results.*
Ethics oversight	*Identify the organization(s) that approved the study protocol.*

Note that full information on the approval of the study protocol must also be provided in the manuscript.

Field-specific reporting

Please select the one below that is the best fit for your research. If you are not sure, read the appropriate sections before making your selection.

☒ Life sciences ☐ Behavioural & social sciences ☐ Ecological, evolutionary & environmental sciences

For a reference copy of the document with all sections, see nature.com/documents/nr-reporting-summary-flat.pdf

Life sciences study design

All studies must disclose on these points even when the disclosure is negative.

Sample size	Sample sizes were selected to reflect typical datasets from single field-of-view, multi-minute single-molecule tracking experiments in living cells, consistent with DeepTRACE's goal of rapid generalisation from small datasets and user training and evaluation from single experiments. Because simulations are processed through a real tracking pipeline and subjected to quality-control criteria (e.g., minimum track length, handling of temporal overlaps), final dataset sizes were determined by the available filtered data. For simulated data, trajectories were generated in batches of 300 or 2,000 molecules, with ~60% retained after filtering. For real data, full fields of view from previously published Pol1 single-molecule tracking experiments (Lagage et al., 2022) were used, with quality control based on track length, truncation, and spatiotemporal overlap. No a priori sample-size calculation was performed. The precise training and evaluation dataset sizes are provided for each dataset.
Data exclusions	Tracking data were filtered automatically as part of quality control, based on minimum track length and automated track truncation/elimination procedures. These processes are described in the Online Methods section, and working examples are included in the software

demonstration package provided for peer reviewers. Individual track examples provided in the Supplementary Figures were selected using DeepTRACE's random track selection tool which we also provide to users and peer-reviewers to ensure an unbiased view of the classification performance across the dataset; exclusions were performed in this selection process when the information being communicated was absent from the random selection (e.g. no changepoint present, all examples displaying only one class), in these cases another randomly-assigned replacement track was requested.

Replication

Model classification performance for different dataset sizes was obtained by training three independently initialised models in each case, with results reported as mean ± standard deviation for F1-score and changepoint error. Models were trained multiple times to ensure that presented results are representative. Permutation importance was computed internally by DeepTRACE via five iterations of random permutation of each feature across all localisations and tracks. To verify reproducibility, we have additionally provided for peer-reviewers a detailed walkthough guide with associated data enabling reproduction of key results and functionality.

Randomization

For each Pol1 experimental data, training and test data were taken from separate datasets obtained in different experimental samples to ensure independence between training and evaluated data. For simulated data, all test data were obtained from fully independent simulations generated with different random seeds. For both simulated and experimental data, shuffling and splitting of training and validation data was performed at the track level prior to subsampling, ensuring the information contained within each remains separate. Downsampling of datasets was performed via random track elimination in a process that was also performed independently with a separate random seed for each replicate. Individual track examples provided in the Supplementary Figures were selected using DeepTRACE's random track selection tool, exclusions were performed in this selection process when the information being communicated was absent from the random selection (e.g. no changepoint present, all examples displaying only one class), in these cases another randomly-assigned replacement track was requested.

Blinding

As human annotation is subject to human judgement and bias, we ensured that all human annotation of training data was performed on datafiles prior to importing ground truth. A software block was also placed on the GUI during annotation, preventing the user from leaving the Human Annotation tab as we were concerned that the user observing the Insights or Track Inspector tools could provide global context which may bias such decision-making.

Reporting for specific materials, systems and methods

We require information from authors about some types of materials, experimental systems and methods used in many studies. Here, indicate whether each material, system or method listed is relevant to your study. If you are not sure if a list item applies to your research, read the appropriate section before selecting a response.

Materials & experimental systems

n/a	Involved in the study
☒	☐ Antibodies
☒	☐ Eukaryotic cell lines
☒	☐ Palaeontology and archaeology
☒	☐ Animals and other organisms
☒	☐ Clinical data
☒	☐ Dual use research of concern
☒	☐ Plants

Methods

n/a	Involved in the study
☒	☐ ChIP-seq
☒	☐ Flow cytometry
☒	☐ MRI-based neuroimaging

Plants

Seed stocks

Report on the source of all seed stocks or other plant material used. If applicable, state the seed stock centre and catalogue number. If plant specimens were collected from the field, describe the collection location, date and sampling procedures.

Novel plant genotypes

Describe the methods by which all novel plant genotypes were produced. This includes those generated by transgenic approaches, gene editing, chemical/radiation-based mutagenesis and hybridization. For transgenic lines, describe the transformation method, the number of independent lines analyzed and the generation upon which experiments were performed. For gene-edited lines, describe the editor used, the endogenous sequence targeted for editing, the targeting guide RNA sequence (if applicable) and how the editor was applied.

Authentication

Describe any authentication procedures for each seed stock used or novel genotype generated. Describe any experiments used to assess the effect of a mutation and, where applicable, how potential secondary effects (e.g. second site T-DNA insertions, mosiacism, off-target gene editing) were examined.
