# Supplementary material for: DeepTRACE brings flexible machine learning to single-molecule track analysis
Source: Commun Biol. 2026 Apr 14;9:812. doi: 10.1038/s42003-026-09899-y (PMC13265943; doi:10.1038/s42003-026-09899-y)
Supplement: Supplementary file 12 — Transparent Peer Review file [file 42003_2026_9899_MOESM12_ESM.pdf]

nature portfolio

Peer Review File

# DeepTRACE brings flexible machine learning to single-molecule track analysis

Corresponding Author: Dr Oliver Pambos

**This file contains all reviewer reports in order by version, followed by all author rebuttals in order by version.**

Version 0:

Reviewer comments:

Reviewer #1

(Remarks to the Author)
In this manuscript the authors present their software package "DeepTRACE" for track segmentation and analysis for data from single-particle tracking experiments. After training on a small dataset of trajectories, customised to match the use-case, the model is shown to outperform state of the art techniques. Through use of a visual interface the program can, supposedly, be easily adjusted to the needs of the user. Unfortunately, since it was not made available for review, I cannot judge the accessibility of this software.

The manuscript is well written and clear. As it provides a convenient tool for researchers working with tracking data, it may be of high interest to the community, justifying its publication in Communications Biology. There are a few minor comments I ask the authors to consider:

(1) In the main text it is unclear how the "inference of global kinetic parameters" (the diffusion coefficient) is performed precisely. This is especially confusing since the model is only introduced as a (piecewise) classification tool. Could you add a more detailed explanation in the main text, right now this is only mentioned near the very end of the method section.

(2) I am somewhat concerned by the use of an ML model with over 100,000 parameters in conjunction with training sets of only a couple of hundred trajectories. Can you comment on the prevalence of overtraining in your experiments and whether a smaller model could be more suitable?

(3) In the conclusion the notion of reinforcement learning is mentioned, however, it is unclear to me how it would be used to improve performance. Could you elaborate on this point?

(4) As it is one of the main selling points, it might be useful to include an example figure of the user interface of DeepTrace in the manuscript.

(5) I find the bibliography a bit limited, it would be useful to provide a more exhaustive list for non-specialist readers. For instance, for single particle tracking, I find Rep Prog Phys 78, 124601 (2015) quite useful. In the context of other machine-learning approaches, Nature Comm 13, 6717 (2022) and 16, 6749 (2025); Biophys J 117, 185 (2019); Nat Met 22, 1091 (2025); PNAS 118, e2104624118 (2021); Nano Lett 24, 3082 (2024) could be mentioned.

As an aside comment: I was curious & checked the name deeptrace, finding that it is already taken by a commercial company, albeit with different capitalisation. There is also DeepTrack by the Volpe group in Gothenburg. Apart from potential legal challenges, maybe this will lead to confusion?

Reviewer #2

(Remarks to the Author)
DeepTRACE is a method by Pambos et al., described in this paper, which allows for context-aware and eep-learning based classification of sub-cellular molecular behaviour of single-particle tracking. The manuscript clearly describes the methodology behind DeepTRACE and provides extensive testing against ground-truth (via simulations) and state-of-the-art alternatives. Additionally, DeepTRACE is tested on experimental data of interesting and relevant data in the world of DNA repair in prokaryotes.

In totality, I believe that DeepTRACE is extremely well-designed, thoroughly tested, and very clearly explained in the manuscript. DeepTRACE is very well thought-out to be of real-world use for researchers in this field. The combination of high-dimensional feature engineering and user-based feature selection (accompanied by computational Feature Ranking methods) is novel and scientifically elegant. Furthermore, the showed results clearly showcase the superiority of DeepTRACE over existing methods. All in all, I believe this manuscript to be of very high quality.

Most of my comments are minor or concern missing information:
* Without access to the software, almost all claims of the paper cannot be accurately reviewed. Especially the multiple references towards 'accessible, user-friendly interface' necessitate review of the software.
* Extended data figure 10 appears to be missing (mentioned on page 30).
* I believe that DeepTRACE inheritly relies on 2-dimensional SPT data, not 3-dimensional. I believe either acknowledging this limitation, or ideally, incorporating the possibility for 3d SPT (only requires minor changes - z pos in the features, and changing some formulas such as the step angles, and apparent diffusion coefficient to be dimension-dependant), would improve the manuscript.
* I think the biggest limit of DeepTRACE is not mentioned: it requires some form of functional annotation (fig1a3). This is done either via simulation or via user-annotation. In cases with novel biology, where it is unsure whether there are multiple functional states, and/or how many, and/or how they behave, this might become problematic. This should either be clearly mentioned, or ideally, some method to select 'statistically significantly different subtrajectories' (or similar) could be included.
* I am a bit confused about the further enhancement of changepoint detection through the increase of class transition rates (lines 106-109, also somewhere in Methods). If I understand this correctly, this effectively increases the transtition rates by ~10-fold, so the network has 'an easier time finding the transitions'? It is unclear why this works (the training data becomes different from inference or real data), why this doesn't over-count transitions, and when/how/why this should or should not be applied.

Overall, I like to reiterate that I believe this is a very good manuscript and method. I can especially appreciate the following:
* fast training/inference
* extremely good documentation of the features
* some user choice of DL model
* Clear emphasis on explanation of important features via the Pearson correlation and SHAP.

Version 1:

Reviewer comments:

Reviewer #1

(Remarks to the Author)
I thank the authors for the detailed responses and the changes in the manuscript. I still find the topic of high relevance and the presented software highly useful. I warmly recommend this revision for publication in Comm Biol.

Reviewer #2

(Remarks to the Author)
My first review round was already positive, and the authors have worked through all my comments nicely and agreeably.

I have reviewed the provided demonstration package, and I must admit that I am thoroughly impressed with the state of the software. It is indeed easy to install/use, but impressively has many features that are outside the core requirements of the method. As such, I see this package to be useful not only for the core DeepTRACE workflow, but also for investigation/visualisation etc for this type of data outside.

**Open Access** This Peer Review File is licensed under a Creative Commons Attribution 4.0 International License, which permits use, sharing, adaptation, distribution and reproduction in any medium or format, as long as you give appropriate credit to the original author(s) and the source, provide a link to the Creative Commons license, and indicate if changes were made.
In cases where reviewers are anonymous, credit should be given to 'Anonymous Referee' and the source.
The images or other third party material in this Peer Review File are included in the article's Creative Commons license, unless indicated otherwise in a credit line to the material. If material is not included in the article's Creative Commons license and your intended use is not permitted by statutory regulation or exceeds the permitted use, you will need to obtain permission directly from the copyright holder.
To view a copy of this license, visit https://creativecommons.org/licenses/by/4.0/

Referee expertise:

Referee #1: biophysics, deep learning

Referee #2: single-molecule microscopy, deep learning

Reviewers' comments:

Reviewer #1 (Remarks to the Author):

In this manuscript the authors present their software package "DeepTRACE" for track segmentation and analysis for data from single-particle tracking experiments. After training on a small dataset of trajectories, customised to match the use-case, the model is shown to outperform state of the art techniques. Through use of a visual interface the program can, supposedly, be easily adjusted to the needs of the user. Unfortunately, since it was not made available for review, I cannot judge the accessibility of this software.

**Response:**

We thank the reviewer for noting this. A demonstration package was uploaded to Zenodo and links/access tokens were forwarded to the editorial office via email immediately after transfer to Communications Biology, which unfortunately appears not to have reached the peer reviewers. We have re-forwarded this email to the handling editor to distribute to the peer reviewers.

The demonstration package consists of,

- A frozen, minimal version of DeepTRACE used to generate the key results and demonstrate the system
- A detailed PDF walkthrough
- Demonstration datasets
- Installation instructions
- Supplementary videos
- Pre-trained models used for key results

This frozen minimal version corresponds exactly to the PDF walkthrough, and to the code used to generate all results in the manuscript. It has been tested on MATLAB R2025a on Windows and MacOS. While the core underlying code remains unchanged, we are currently collaborating with other labs to expand compatibility, remove bugs, improve stability, and support additional microscope hardware and file formats prior to public release; however, all analyses described in the manuscript are fully reproducible using this frozen version.

**Note from the editor: Instead of forwarding the email I have put all the information on an additional file in the system. I have also uploaded another file sent by the author.** In the last round, the email with this information was forwarded when we already had received a review, which is why we did not send it to the reviewers.

The manuscript is well written and clear. As it provides a convenient tool for researchers working with tracking data, it may be of high interest to the community, justifying its publication in Communications Biology. There are a few minor comments I ask the authors to consider:

(1) In the main text it is unclear how the "inference of global kinetic parameters" (the diffusion coefficient) is performed precisely. This is especially confusing since the model is only introduced as a (piecewise) classification tool. Could you add a more detailed explanation in the main text, right now this is only mentioned near the very end of the method section.

**Response:**

We thank the reviewer for highlighting the need to clarify this point, which was initially omitted due to space limitations. We have now expanded the main text (lines 62 - 65) to explain that inference of diffusion coefficients is performed by MSD-lag time analysis on subtracks aggregated by class following segmentation.

(2) I am somewhat concerned by the use of an ML model with over 100,000 parameters in conjunction with training sets of only a couple of hundred trajectories. Can you comment on the prevalence of overtraining in your experiments and whether a smaller model could be more suitable?

**Response:**

We thank the reviewer for raising this concern, and we agree that in some scenarios large models trained on small datasets can result in overfitting. Overfitting in DeepTRACE is controlled through regularisation (including dropout), and early stopping which uses a validation split that is performed at the track level prior to subsampling. DeepTRACE displays validation and training loss to the user graphically during the training process, and we did not observe any significant divergence of these losses that would be characteristic of overfitting. As recurrent models share each weight across all timesteps, the parameter count tends to overstate their effective capacity, reducing the tendency to memorise individual sequences. The effective amount of training information is determined by the many timesteps across all tracks rather than the small number of tracks themselves. As an additional check, we trained a model with early stopping disabled, and with a greatly reduced learning rate schedule, and in this case clear overfitting was observed, confirming that the safeguards operate as expected. Taken together, these results indicate that the models used are not overfitting the available data.

During development, we also trained smaller models (~5,000 parameters) by reducing the number of RNN hidden units, and observed similar training time and performance to the final models (>100,000 parameters), but crucially far more reliable training in the latter. We interpret this using recent work on the lottery ticket hypothesis (arXiv:1803.03635) which shows that large networks, including LSTMs (arXiv:1906.02768), contain much smaller well-initialised subnetworks. For this reason, we use larger models during training by default, as they make it far more likely that such a trainable subnetwork exists and can be found reliably, leading to more stable training. These sparse subnetworks can in principle be obtained in smaller models, either

by pruning the original model while keeping the early training weights (late rewinding), or by repeated training runs of a smaller architecture until a similarly well-initialised ‘winning ticket’ is encountered.

We do not see a use case for implementing such a pruning system for users performing track classification tasks as inference even with the largest models runs in a few hundred milliseconds, with an additional 1-2 seconds for the consensus voting system (a model-independent post-processing step), so larger models have no practical impact on inference speed or hardware requirements.

To test whether similar performance is possible using more compact models, we retrained much smaller model architectures (2,172 - 4,817 parameters; median 2,273) across all simulation types and real experimental datasets, and found that these compact models often require multiple training runs to reach the same performance as the larger models, consistent with the lottery ticket hypothesis.

We have updated the manuscript and figures to illustrate the results obtained with these smaller models (see Figures 2b, 2d, all subfigures of Extended Data Figures 4 and 5, and all subfigures of Supplementary Figures 1 to 4).

To avoid cherry-picking tracks, we identified the original unique track identifiers for all track examples in the original manuscript, and re-annotated the same tracks using the smaller models, allowing direct comparison of the new results; all individual track examples in the manuscript now show annotations from these compact models. The methods section has been updated to clarify the thinking around model size and overfitting (lines 539, 541 - 548, 557, 558). We have also updated figure legends throughout the manuscript with these model sizes.

(3) In the conclusion the notion of reinforcement learning is mentioned,
however, it is unclear to me how it would be used to improve performance.
Could you elaborate on this point?

**Response:**

We thank the reviewer for this helpful comment. We have expanded the conclusion to clarify our future development plans. Given the limited dataset sizes typical in single-molecule tracking and the modest throughput of human annotation, we plan to implement an offline user-feedback system that enables adaptive fine-tuning from human input. This will allow users to flag or correct mis-classified track segments during inspection, and these annotations will be incorporated into subsequent retraining rounds to improve performance on challenging behaviours. The conclusion has been updated accordingly (lines 132 - 135).

(4) As it is one of the main selling points, it might be useful to include
an example figure of the user interface of DeepTrace in the manuscript.

**Response:**

This is a good suggestion. We have included in Figure 1 a screenshot of the software in operation while performing human annotation on real Pol1-JF549 tracking data. The figure also displays (in the background) data visualisations produced by DeepTRACE as an illustration of the graphical interface.

(5) I find the bibliography a bit limited, it would be useful to provide a more exhaustive list for non-specialist readers. For instance, for single particle tracking, I find Rep Prog Phys 78, 124601 (2015) quite useful. In the context of other machine-learning approaches, Nature Comm 13, 6717 (2022) and 16, 6749 (2025); Biophys J 117, 185 (2019); Nat Met 22, 1091 (2025); PNAS 118, e2104624118 (2021); Nano Lett 24, 3082 (2024) could be mentioned.

**Response:**

We thank the reviewer for kindly providing these additional references. We have now incorporated all of them, including those published after the initial submission. In response to the reviewer's suggestion, we added two brief notes to the introduction: one summarising classical statistical approaches for single-molecule track analysis (e.g., MSD-based methods, HMMs, and Bayesian inference), and another summarising recent machine-learning approaches for diffusion classification and track segmentation (lines 14 - 15). These additions should provide non-specialist readers with a clearer, and more complete picture of the broad landscape of single-molecule track analysis. Additionally, we have briefly mentioned the confidence estimation methods discussed in Nature Comm 13, 6717 (2022) as a possible future direction to further expand DeepTRACE's interpretability features (lines 135 - 137), and Nano Lett 24, 3082 (2024) as an excellent example of multi-modal tracking involving anisotropy readouts that could be handled directly by DeepTRACE's arbitrary feature input (lines 127 - 128).

As an aside comment: I was curious & checked the name deeptrace, finding that it is already taken by a commercial company, albeit with different capitalisation. There is also DeepTrack by the Volpe group in Gothenburg. Apart from potential legal challenges, maybe this will lead to confusion?

**Response:**

We thank the reviewer for raising this point. Several commercial products share the name "DeepTrace" with different capitalisations, but they operate in unrelated domains (e.g., fraud detection, cybersecurity, medical prognosis) and have entirely separate user bases from our academic tool. "DeepTRACE" is also distinct from the academic software "DeepTrack"; we use full capitalisation of "TRACE" to avoid any confusion. There is no trademark conflict, and we therefore retain the name.

Reviewer #2 (Remarks to the Author):

DeepTRACE is a method by Pambos et al., described in this paper, which allows for context-aware and eep-learning based classification of sub-cellular molecular behaviour of single-particle tracking. The manuscript clearly describes the methodology behind DeepTRACE and provides extensive testing against ground-truth (via simulations) and state-of-the-art alternatives. Additionally, DeepTRACE is tested on experimental data of interesting and relevant data in the world of DNA repair in prokaryotes.

In totality, I believe that DeepTRACE is extremely well-designed, thoroughly tested, and very clearly explained in the manuscript. DeepTRACE is very well thought-out to be of real-world use for researchers in this field. The combination of high-dimensional feature engineering and user-based feature selection (accompanied by computational Feature Ranking methods) is novel and scientifically elegant. Furthermore, the showed results clearly showcase the superiority of DeepTRACE over existing methods. All in all, I believe this manuscript to be of very high quality.

Most of my comments are minor or concern missing information:
* Without access to the software, almost all claims of the paper cannot be accurately reviewed. Especially the multiple references towards 'accessible, user-friendly interface' necessitate review of the software.

**Response:**

We thank the reviewer for noting this. A demonstration package was uploaded to Zenodo and links/access tokens were forwarded to the editorial office via email immediately after transfer to the journal, which unfortunately appears not to have reached the peer reviewers. Please see a more complete response to the same point made by Reviewer 1.

**Note from the editor: Instead of forwarding the email I have put all the information on an additional file in the system. I have also uploaded another file sent by the author.** In the last round, the email with this information was forwarded when we already had received a review, which is why we did not send it to the reviewers.

* Extended data figure 10 appears to be missing (mentioned on page 30).

**Response:**

We apologise for this oversight; the text has been updated to indicate that the correct figure is Supplementary Fig. 6. This change can be found on Page 31, Line 534.

* I believe that DeepTRACE inheritly relies on 2-dimensional SPT data, not 3-dimensional. I believe either acknowledging this limitation, or ideally, incorporating the possibility for 3d SPT (only requires minor changes - z pos in the features, and changing some formulas such as the step angles, and apparent diffusion coefficient to be dimension-dependant), would improve the manuscript.

**Response:**

We thank the reviewer for raising this important point. DeepTRACE is currently implemented for two-dimensional SPT data, which is the dominant format in single-molecule studies of bacteria and most live-cell applications. We agree that extending the system to full 3D tracks would be valuable.

Although DeepTRACE can already import any arbitrary numerical feature in the tracking file (including a z-coordinate) for model training, full integration of 3D information into the engineered feature set and downstream analysis requires substantial development, robust testing, and careful evaluation of consequences (e.g. the influence of different localisation precision in lateral

and axial directions). As the reviewer notes, several engineered features (e.g. step angles, geometric descriptors) and analysis modules (e.g. diffusion estimation, spatial mapping) must be generalised to three dimensions and validated on real 3D tracking data.

We aim to implement this extension in a future release. The manuscript has been updated (lines 131 - 132) to acknowledge that the present work applies to 2D trajectories, with support for 3D modalities planned in a future version of DeepTRACE.

* I think the biggest limit of DeepTRACE is not mentioned: it requires some form of functional annotation (fig1a3). This is done either via simulation or via user-annotation. In cases with novel biology, where it is unsure whether there are multiple functional states, and/or how many, and/or how they behave, this might become problematic. This should either be clearly mentioned, or ideally, some method to select 'statistically significantly different subtrajectories' (or similar) could be included.

**Response:**

The reviewer is correct that DeepTRACE's supervised approach requires functional annotation of training data. In the original text we stated that DeepTRACE can obtain labels from human annotation or simulations, but we did not clarify that these labels are required; we have updated the manuscript to explicitly state that (i) this is a supervised approach that requires such labels, and (ii) annotation can be more difficult when working with unexpected or previously unseen biological behaviours (lines 26 – 28, 30 – 31).

We agree that identifying regions of statistically distinct behaviours prior to functional annotation is valuable; however, unsupervised filtering prior to model training introduces several challenges. First, if an upstream filtering algorithm selects subtracks, the model risks simply learning the behaviour of that algorithm rather than the underlying biology.

Second, any algorithm that selects only the most easily separable regions would tend to exclude subtle or transitional behaviours, which are exactly the examples needed for a supervised model to learn robust decision boundaries. We are concerned that excluding them reduces the diversity of the training distribution, which may limit the classifier's performance to learn from the full range of behaviours. It is our intention instead that the manual annotation approach be driven primarily by human observation of the behaviours, potentially assisted by perturbation selected from the user's biological domain knowledge which would vary in nature between experiments, making the behaviours clearer without significantly modifying the features used for classification. The existing Explainability and Discovery tools may assist users in refining or interpreting state definitions once an initial model has been trained, and the class-based analysis tools are designed to characterise those new states, once identified. While unsupervised subtrack filtering is beyond our current scope, we agree that it is an interesting direction that merits exploration in future versions of the software.

* I am a bit confused about the further enhancement of changepoint detection through the increase of class transition rates (lines 106-109, also somewhere in Methods). If I understand this correctly, this effectively increases the transtition rates by ~10-fold, so the network has 'an easier time finding the transitions'? It is unclear why this works (the training data becomes

different from inference or real data), why this doesn't over-count transitions, and when/how/why this should or should not be applied.

**Response:**

We thank the reviewer for the opportunity to clarify this point. In this simulation we increased the transition rate while keeping the underlying behaviours unchanged. Increasing the number of transitions in the training data allows DeepTRACE to learn a more accurate internal representation of what a genuine changepoint looks like: specifically, the behaviours encoded in the temporal evolution of features, and the relationships between features that are conserved between the two datasets. The resulting model recognises true changepoints more reliably in the test data as evidenced by the improved classification metrics (changepoint error, Extended Data Fig. 9a). This type of perturbation is particularly useful in experimental scenarios in which a rare event needs to be classified in unseen data; we also now include an example of an experimental scenario in which this can occur.

Unlike models such as ResAnDi2, DeepTRACE does not contain a dedicated module for changepoint detection; instead changepoints are identified directly from the post-classified sequences. Adding additional examples of changepoints results in the model more robustly identifying the true changepoints, rather than learning (and reproducing) an expected rate imprinted during training. This illustrates that DeepTRACE has learned a more accurate internal representation of a changepoint, and successfully identifies these events in the unperturbed dataset better than an identical model trained on the same quantity of unperturbed tracks.

We have expanded the main text to clarify these points (lines 115 - 122).

Overall, I like to reiterate that I believe this is a very good manuscript and method. I can especially appreciate the following:
* fast training/inference
* extremely good documentation of the features
* some user choice of DL model
* Clear emphasis on explanation of important features via the Pearson correlation and SHAP.
